# An Unusual Presentation of B-Cell Lymphoma as a Large Isolated Epiglottic Mass: Case Report and Literature Review

**DOI:** 10.1155/2016/9787432

**Published:** 2016-04-26

**Authors:** Changxing Liu, Sean Delaney, Tamara N. Brown

**Affiliations:** Department of Otolaryngology, Keck School of Medicine of University of Southern California, Los Angeles, CA 90033, USA

## Abstract

Extranodal presentation of B-cell lymphoma is uncommon. Isolated primary epiglottic B-cell lymphoma is even rarer. To our knowledge, there has been only one description of isolated B-cell lymphoma presenting as a large epiglottic mass. We report an unusual type of B-cell lymphoma of the epiglottis, as it could not be subtyped based on routine staining and hybridization. The lymphoma presented as a large isolated globular mass pedicled to the epiglottis, occupying most of the oropharynx, but did not have any ball-valving effect or increased respiratory efforts. Initial radiographic findings were nonspecific. The diagnosis of B-cell lymphoma was determined by transoral incisional biopsy under local anesthesia. The condition was treated successfully with chemoradiation. The current standard of treatment for high grade B-cell lymphoma is concurrent chemoradiotherapy, with excellent prognosis. Although rare, B-cell lymphoma should be considered when investigating pedunculated hypopharyngeal masses.

## 1. Introduction

Primary extranodal lymphoma most frequently arises from the gastrointestinal tract, with the head and neck being the second most frequently involved region, accounting for 2.5% of all lymphoma cases [1, 2]. Isolated presentation in the larynx or hypopharynx is rare, accounting for 1% of all laryngeal tumors, with the B-cell phenotype as the predominant [3]. To date, there is only one report of B-cell lymphoma presenting as an isolated epiglottic mass [4]. We present a case of primary B-cell lymphoma of the epiglottis that arose in a 60-year-old female as a sessile mass on the laryngeal surface of the epiglottis. Her pathology showed an unusual mixed grade B-cell lymphoma, with both low and high grade features, atypical staining patterns, and in situ hybridization findings. Although squamous cell carcinoma is the most common etiology for an epiglottic tumor, lymphoma is an important differential consideration because its first-line treatment is chemoradiation, instead of surgery. Although excisional biopsy or resection is tempting, our patient was treated with chemoradiation by following the current protocols, and she had a very good response. In the paper we share the second reported case of B-cell lymphoma presenting as an isolated epiglottic mass and conduct a review of the current literature.

## 2. Case Report

A 60-year-old Caucasian female presented with a 2-month history of progressive dysphagia, hoarseness, and globus sensation, accompanied by a 10-pound weight loss over this period. At time of presentation, she also reported exertional dyspnea. She denied nausea, vomiting, fever, hemoptysis, or hematemesis. Her only other medical comorbidity was hypertension, which was well controlled. She denied smoking, drinking, or related family history of malignancy.

**Figure 1:**
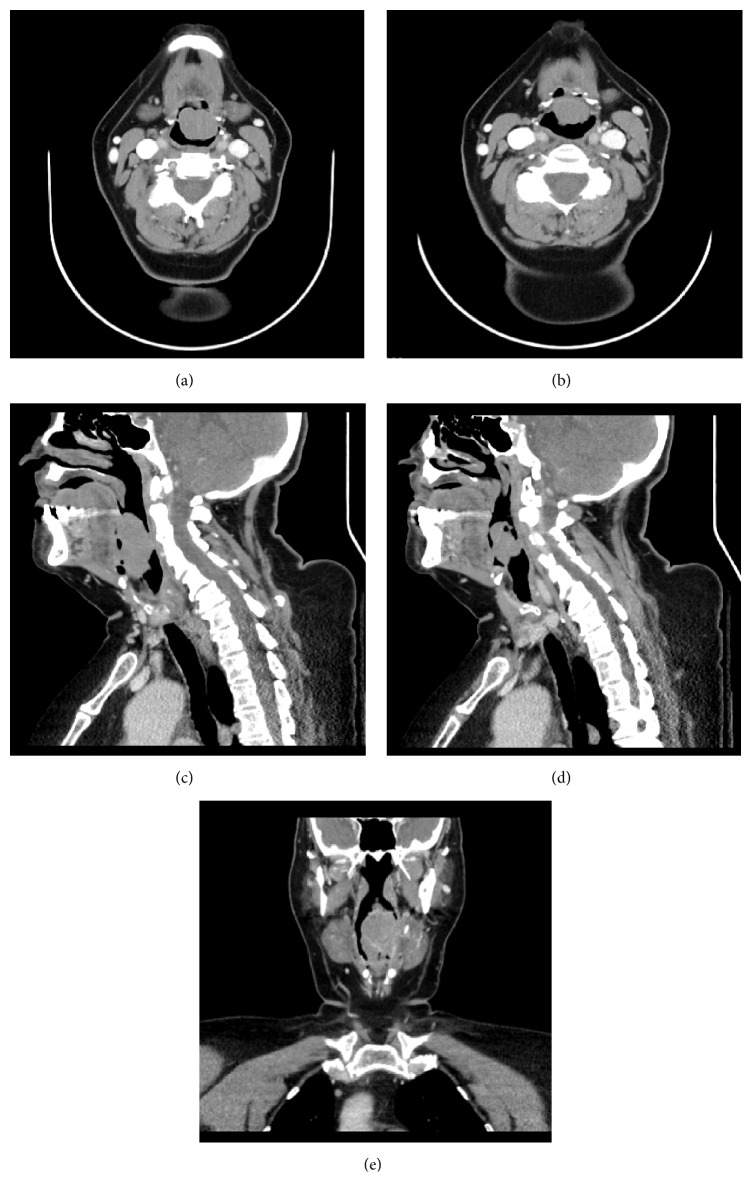
Axial CT neck (a and b), sagittal CT neck (c and d), and coronal CT neck (e), with contrast, show the anteriorly sessile soft tissue mass arising from the laryngeal surface of epiglottis, with relatively stable position. The epiglottis has no submucosal extension.

A CT neck with contrast revealed a large sessile, isointense mass on the laryngeal surface of epiglottis measuring 4.7 cm in its greatest dimension, with no submucosal extension or cervical lymphadenopathy (see Figure 1).

**Figure 2:**
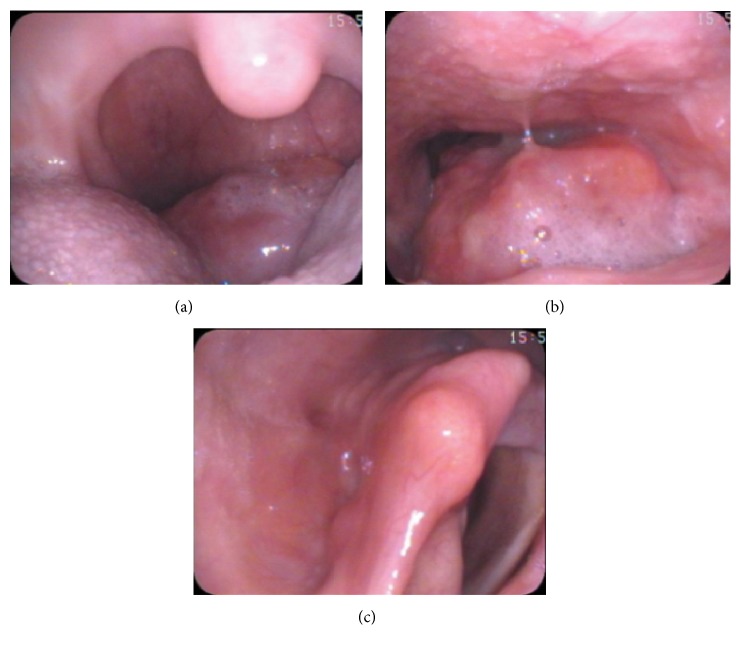
Direct visualization of the mass through mouth (a) and flexible fiberoptic laryngoscopy shows the mass extending to oropharynx (b), but the glottis was not involved and was visualized when passing the scope around the mass (c).

The patient was subsequently referred to the otolaryngology service for further evaluation. On examination she had a muffled voice and minimal inspiratory stridor without respiratory distress. On palpation, she had normal external laryngeal landmarks and no palpable neck masses. Using a tongue depressor, a large mass could be seen in the oropharynx behind the upper edge of epiglottis. Flexible fiberoptic examination confirmed an exophytic mass in oropharynx that was sessile to the laryngeal surface of the epiglottis and occupied most of the oropharynx. Just beyond the mass, the hypopharynx and laryngeal inlet were normal in appearance (see Figure 2).

**Figure 3:**
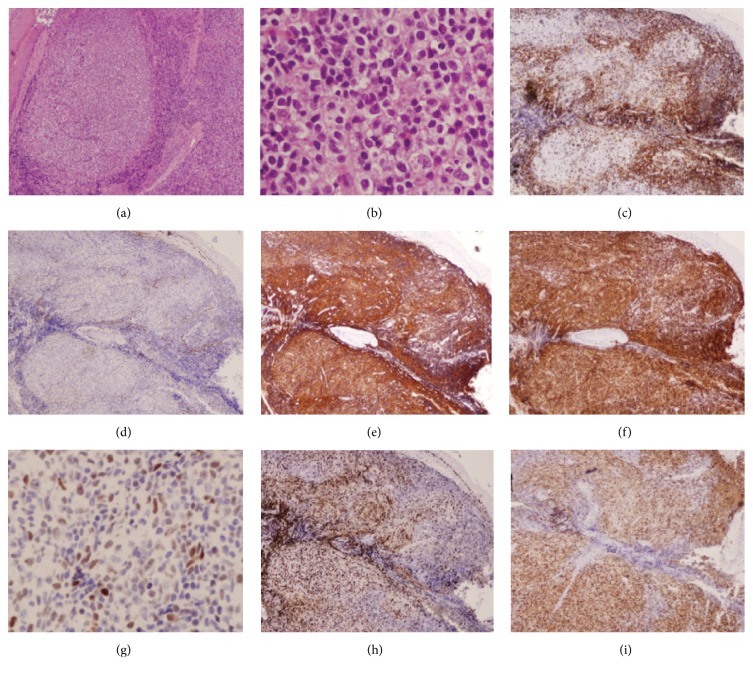
Permanent sections showing histology of B-cell lymphoma; low power (a) and high power (b) show the typical histological structure. Further immunostainings of the mass for typical lymphoma markers, including CD3 (c), CD10 (d), CD20 (e), BCL2 (f), BCL6 (g), Ki67 (h), and MUM1 (i).

A transoral biopsy of the mass was conducted under local anesthesia, since it was easily visualized through her mouth and the mass was stable without glottis obstruction. The biopsy revealed an unusual B-cell lymphoma subtype (see Figure 3), based on immunostaining and in situ hybridization (Table 1).

CT of chest, abdomen, and pelvis was negative for additional lymphadenopathy. Bone marrow aspiration and biopsy showed no evidence of bone marrow involvement. These findings were consistent with a diagnosis of diffuse large B-cell (high grade; also she has intermingled low grade areas) non-Hodgkin lymphoma of the larynx, stage IE, with an R-IPI score of 1 (age > 60, ECOG 0–2, normal LDH of 209 U/L, 0-1 extranodal sites, and stage I/II disease). The patient was subsequently treated with definitive chemotherapy and radiation.

**Figure 4:**
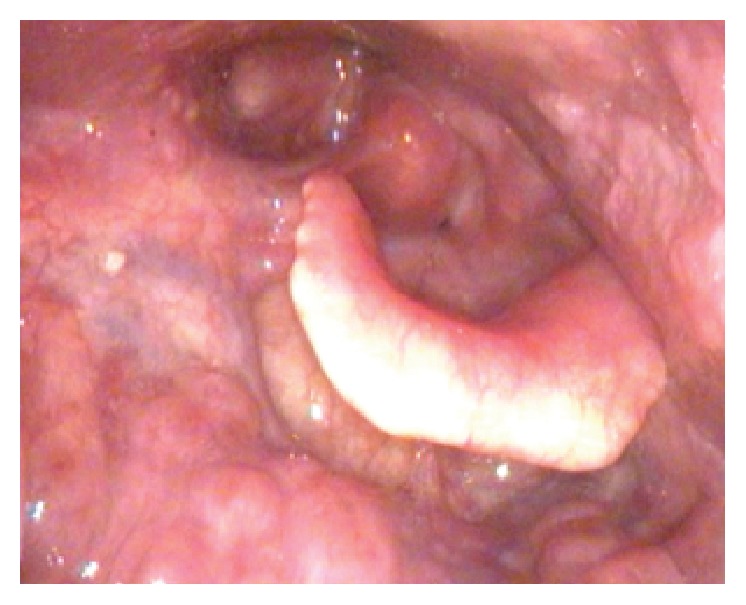
Laryngoscopy was performed three months posttreatment. There was no evidence of residual tumor.

She was given three cycles of R-CEOP (rituximab, cyclophosphamide, etoposide, vincristine, and prednisone) followed by involved field radiotherapy to the neck with 40 Gy radiation. The patient was seen in clinic 8 weeks later with complete resolution of dysphonia and dysphagia. Repeat fiberoptic examination demonstrated complete resolution of the epiglottic mass without evidence of recurrent or residual tumor. She was seen again four months later with normal voice and remained disease free on examination (Figure 4). Her PET scan 3 months after treatment was negative.

## 3. Discussion

Extranodal non-Hodgkin lymphomas limited to the larynx are rare, accounting for less than 1% of all laryngeal neoplasms. The literature review shows that all sites inside the larynx can be involved. The most commonly involved site is the supraglottic region (47%) [5]. Fewer than 100 cases of lymphoproliferative tumors arising from the larynx, including both NHL and immunosuppression-related lymphoproliferative diseases, have been previously reported in the literature [6]. The mean age at diagnosis is 70 years with variable sex-ratio in different series [7, 8]. Our patient was a 60-year-old female. Symptoms of laryngeal lymphoma typically include dysphonia, hoarseness, dysphagia, and cervical lymphadenopathy [9–11]. There are certain imaging characteristics that should suggest lymphoma. Laryngeal NHL is a tumor that usually has a large submucosal component, which should alert the radiologist to the possibility of NHL [6].

Despite being a part of the supraglottis, the epiglottis is rarely reported as the primary site of laryngeal lymphoma. Only select few cases of NK/T-cell lymphomas were reported as originating from the epiglottis [12], and less than 3 cases of MALT lymphoma were associated with the epiglottis [8, 13]. In our patient, special lymphoma markers were stained for subgroup analysis, but the findings were inconclusive. The final pathologic diagnosis was that of an unusual high grade type of B-cell lymphoma. Our patient has a typical nonnecrotic supraglottic lesion with homogeneous enhancement, which is characteristic of lymphoma [5], but the lesion did not demonstrate a submucosal component or any submucosal extension, which is characteristic of epiglottic lymphomas.

Histologically, primary laryngeal lymphoma is more commonly of B-cell origin, though some T-cell and NK cell lymphomas were found before [12]. A panel of commonly used markers for final subtype diagnosis includes leukocyte common antigen (LCA), B-cell markers (CD20 and CD79a), T-cell markers (CD3 and CD5), and other markers like CD23, BCL2, BCL6, and CD10. Our patient had an unusual B-cell lymphoma, which exhibits both low grade and high grade features. Her H&E sections showed an abnormal nodular lymphoid infiltrate composed primarily of medium-sized cells. Many of the cells have cleaved nuclei, granular chromatin, inconspicuous nucleoli, and abundant clear cytoplasm. The cytomorphologic differential diagnosis includes follicular lymphoma and extranodal marginal zone lymphoma (MALT lymphoma). The immunophenotype (CD10−/BCL6+/MUM1+) supported a nongerminal center B-cell origin, raising concern for a diffuse large B-cell lymphoma (DLBCL). However, the relatively low Ki67 proliferation rate (~10%) in the majority of the lymphoid infiltrate ruled against the diagnosis of DLBCL, which usually has proliferation rates of 40% or greater. A focus with many apoptotic cells does appear to demonstrate higher Ki67 staining, but assessment is precluded by extensive crush artifact. EBER (Epstein-Barr encoding region) ISH was negative. Overall, the nongerminal center B-cell like immunophenotype is suspicious for DLBCL; however, the cytomorphology and relatively low proliferation rate argue against a definitive diagnosis of DLBCL. The negative cyclin D1 argued against a mantle cell lymphoma. Low grade follicular lymphoma was a consideration; however, the negative CD10 and positive MUM1 would be unusual for this entity. Similarly, the BCL6 and MUM1 positivity were not typical for a MALT lymphoma.

The main modalities of treatment for B-cell lymphoma are radiotherapy alone or radiotherapy in combination with chemotherapy [14–17]. Our patient underwent 3 cycles of R-CEOP followed by external beam radiotherapy. Surgical intervention, based on tracheotomy or on laser debulking, is only usually required in patients presenting with acute airway obstruction [15, 18, 19]. Combined chemoradiotherapy is the preferred treatment modality, especially for high grade lymphomas, and provides excellent outcomes [15, 16, 18, 20, 21]. As for our case, since the patient does not have any obvious submucosal component or any submucosal extension, surgical excision may have been considered should she develop more aerodigestive obstructive symptoms. Our patient demonstrated complete resolution of the tumor with convention chemoradiotherapy. Three months after the completion of her treatment, her PET scan was negative.

## 4. Conclusion

Primary laryngeal lymphoma is a rare entity, and primary epiglottic lymphoma is even rarer. It does not have the typical submucosal component or submucosal extension that other laryngeal lymphomas usually exhibit. Computed tomography can demonstrate nonspecific homogenous enhancement. Direct biopsy makes the diagnosis. Combined chemoradiotherapy is the preferred modality of treatment. We report a rare case of unusual presentation of B-cell lymphoma as a primary pedunculated epiglottic mass. With extensive immunostaining, we still cannot determine it as a known subtype. This can be a mixture type or a new subtype. Although rare, B-cell lymphoma should be considered when investigating pedunculated hypopharyngeal masses.

## Figures and Tables

**Table 1:** Details of the immunostaining and hybridization: immunohistochemistry^*∗*^.

	Immunostaining/ISH	
1	CD3	Scattered small T-lymphocytes positive
2	CD10	Lymphoma negative
3	CD20	Lymphoma positive
4	CD21	Rare partially disrupted follicular dendritic cell networks highlighted
5	CD23	Negative
6	CD43	Negative with scattered small lymphocytes positive
7	BCL2	Positive
8	BCL6	Positive (45% in a cell count of 200)
9	MUM1	Positive
10	Cyclin D1	Negative
11	C-MYC	Negative
12	Kappa	Extremely rare plasma cells positive
13	Lambda	Extremely rare plasma cells positive
14	Ki67	Low proliferation rate in majority of lymphoid infiltrate (approximately 10% cells in cycle overall); one focus with extensive crush artifact demonstrates increased proliferation rate (~20–30%)
15	EBER ISH	Negative

^*∗*^Performed at LAC + USC Medical Center Immunohistochemistry Laboratory.
